# Pharmacokinetic and neuroimmune pharmacogenetic impacts on slow-release morphine cancer pain control and adverse effects

**DOI:** 10.1038/s41397-024-00339-w

**Published:** 2024-06-01

**Authors:** Daniel T. Barratt, Pål Klepstad, Ola Dale, Stein Kaasa, Andrew A. Somogyi

**Affiliations:** 1 Discipline of Pharmacology, Faculty of Health and Medical Sciences, University of Adelaide, Adelaide, Australia; 2 Discipline of Physiology, Faculty of Health and Medical Sciences, University of Adelaide, Adelaide, Australia; 3 Department of Circulation and Medical Imaging, Norwegian University of Science and Technology, Trondheim, Norway; 4 Department of Anaesthesiology and Intensive Care Medicine, St Olavs University Hospital, Trondheim, Norway; 5 Department of Oncology, Institute of Clinical Medicine, University of Oslo, Oslo, Norway; 6 Department of Clinical Pharmacology, Royal Adelaide Hospital, Adelaide, Australia

**Keywords:** Signs and symptoms, Predictive markers, Genetics research, Predictive markers, Risk factors

## Abstract

The aim was to determine if opioid neuroimmunopharmacology pathway gene polymorphisms alter serum morphine, morphine-3-glucuronide and morphine-6-glucuronide concentration-response relationships in 506 cancer patients receiving controlled-release oral morphine. Morphine-3-glucuronide concentrations (standardised to 11 h post-dose) were higher in patients without pain control (median (interquartile range) 1.2 (0.7–2.3) versus 1.0 (0.5–1.9) μM, *P* = 0.006), whereas morphine concentrations were higher in patients with cognitive dysfunction (40 (20–81) versus 29 (14–60) nM, *P* = 0.02). TLR2 rs3804100 variant carriers had reduced odds (adjusted odds ratio (95% confidence interval) 0.42 (0.22–0.82), *P* = 0.01) of opioid adverse events. IL2 rs2069762 G/G (0.20 (0.06-0.52)), BDNF rs6265 A/A (0.15 (0.02–0.63)) and IL6R rs8192284 carrier (0.55 (0.34–0.90)) genotypes had decreased, and IL6 rs10499563 C/C increased (3.3 (1.2–9.3)), odds of sickness response (*P* ≤ 0.02). The study has limitations in heterogeneity in doses, sampling times and diagnoses but still suggests that pharmacokinetics and immune genetics co-contribute to morphine pain control and adverse effects in cancer patients.

## Introduction

Cancer patients experience many symptoms associated with not only their disease but also specific treatments. One of the common symptoms is moderate to severe pain, with opioids the mainstay of cancer pain management. Opioids effectively manage pain in over 80% of cancer patients, however 20% will experience intolerable adverse effects that reduce their quality of life and cause suffering [1].

For patients who can take oral medications, controlled-release oral morphine is frequently used but, like all opioids, wide interindividual variability in response and adverse effects is observed [1] necessitating careful dose individualisation. This variability can have a combination of pharmacokinetic (drug and metabolite exposures) and pharmacogenetic (enzymes, transporters, signalling pathways) components.

Morphine metabolites (morphine-3-glucuronide (M3G) and morphine-6-glucuronide (M6G)) are pharmacologically active. M6G is a potent mu agonist which can cause respiratory depression especially in those with reduced kidney function, with no innate immune activation properties. Conversely, M3G has no mu opioid receptor activity but can potently activate the innate immune system by binding to MD2, the accessory protein for TLR4 [2–4]. M3G has been implicated in morphine-induced CNS toxicity (seizures, cognitive impairment) at high doses [5, 6]. In addition to pain, cancer patients also experience other common symptoms such as nausea, fatigue and depression which are characteristic of cytokine-induced sickness responses, with cognitive dysfunction observed in cancer patients undergoing inflammatory cytokine immunotherapy [7]. Thus, any assessment of morphine efficacy and adverse effects needs to take into consideration the pharmacology of both morphine and its metabolites [8, 9].

The European Pharmacogenetic Opioid Study (EPOS) was a multinational collaborative effort to identify factors, particularly genetic, that determine opioid requirements for moderate-severe cancer pain [10]. Given the neuroimmunopharmacology of morphine and its metabolites, the aim of this study was to investigate if common polymorphisms in genes involved in innate immune activation, inflammatory signalling, neuronal pathways and transporter genes indirectly alter the serum morphine, M3G and M6G concentration-response relationships for pain control, cognitive dysfunction, and adverse symptom complaint (sickness response and opioid side-effects), in EPOS cancer pain patients on slow-release oral morphine.

## Methods

### EPOS subjects and data

EPOS [10] is a multicentre collaborative study of 2294 cancer patients with a malignant disease treated with an opioid for moderate to severe pain (step III of WHO treatment ladder) [11]. All patients provided written informed consent and the protocol was approved at each study centre’s local ethics committee. Of EPOS participants, 558 were treated with oral slow-release morphine. After excluding 15 non-Caucasian participants, a further 37 patients had no data for any outcome measure, leaving 506 with data required for analysis of at least one outcome measure (465 for all outcome measures) who were included in the final study analyses.

A complete list of patient data taken as part of the original EPOS study is available [10]. These data include serum morphine, M3G and M6G concentrations (Table 1), average pain using the Brief Pain Inventory (BPI) [12] and cognitive function using the Mini-Mental State Examination (MMSE) [13, 14]. European ancestry subgroups were also previously determined [15].

**Table 1:** Patient characteristics and investigated non-genetic variables (for *n* = 506 included in outcome analyses).

Variable	*n*	Median (interquartile range: range) or counts.	Analysis notes^b^
Age	505	64 (56–72: 27–91)	Square transformed
Sex	506	277 Male / 229 Female	
Treatment centre country	506	Switzerland = 47; Germany = 68; Denmark = 12; United Kingdom = 60; Iceland = 91; Italy = 31; Norway = 158; Sweden = 39.	
BMI (kg/m^2^)	493	24 (21–27; 14–44)	Log transformed
Serum albumin concentration (g/L)	487	33 (28-37: 10-56)	Square transformed
Serum C-reactive protein concentration (mg/L)	502	≤ 40 mg/L = 289 > 40 mg/L = 213	
Creatinine clearance (Cockcroft–Gault [46]: mL/min)	488	91 (68–117: 9–277)	Square root transformed
Kidney Disease	506	21	
Time on opioids (days)	475	46 (16–149: 1–3725)	Log transformed
Scheduled morphine dose (mg/day)^a^	506	60 (40–120: 10–1600)	
Serum morphine concentration (nM)	503	39 (16–91: 4–1437)^c^; *n* = 30 < LLOQ	
Serum morphine-3-glucuronide concentration (μM)	503	1.27 (0.64–2.47: 0.04–40.4)^c^; *n* = 7 < LLOQ	
Serum morphine-6-glucuronide concentration (nM)	503	245 (120–484: 5–8949)^c^; *n* = 7 < LLOQ	
Time between opioid dose and serum sample (minutes)	471	640 (308–680: 5–1410)	
Standardised serum morphine concentration (nM)	450	31 (15–63: 3–759)	λ = −0.1 transformed
Standardised serum morphine-3-glucuronide concentration (μM)	469	1.08 (0.57–2.13: 0.03–27.9)	Log-transformed
Standardised serum morphine-6-glucuronide concentration (nM)	469	221 (110–425: 4–6264)	Log-transformed
Cancer diagnosis	506	Haematological = 29; Breast = 75; Prostate = 67; Urological = 31; Lung = 102; Gastrointestinal = 90; Female reproductive = 21; Sarcoma = 8; Head and neck = 27; Pancreatic = 6; Skin = 12; Liver = 0; Mesothelioma = 5; Unknown origin = 18; Other = 15.	
Metastases	506	Any = 429; Liver = 133; Bone = 245; CNS = 41; Lung = 110; Other = 178.	
Co-medications in previous 24 h	506	Any = 498; Breakthrough opioid = 185; Gabapentin = 80; Weak opioid = 8; Systemic glucocorticoid = 255; Paracetamol = 196; Benzodiazepine = 122; NSAID = 156; Hypnotic = 136; Antidepressant = 120; Laxative = 328; ACE inhibitor = 44; Antiemetic = 190; Ranitidine = 329.	
Pain category	506	Visceral = 57; Bone and soft tissue (deep somatic) = 237; Neuropathic = 21; Mixed = 181; Unknown = 10.	
Pain location	493	Head = 65; Thoracic/upper abdominal = 161; Pelvic = 331; Back = 172; Upper extremity = 77; Lower extremity = 143.	
Karnofsky Performance Status Scale [47]	504	60 (50–73: 20–90)	
EORTC QLQ-C30: Nausea and vomiting symptom scale	485	<50 = 388 ≥ 50 = 97	
EORTC QLQ-C30: Constipation symptom scale	485	<50 = 278 ≥ 50 = 207	
EORTC QLQ-C30: “Were you tired?”	479	“Not at all” or “A little” = 161 “Quite a bit” or “Very much” = 318	
EORTC QLQ-C30: “Did you feel depressed?”	481	“Not at all” or “A little” = 339 “Quite a bit” or “Very much” = 142	

^a^Not included as a non-genetic variable. ^b^Data transformations to a normal distribution for further analysis [λ represents Box-Cox transformation: (x^λ^-1)/λ]. ^c^Medians and ranges are for concentrations >LLOQ. BMI: body mass index. EORTC QLQ-C30: European Organisation for Research and Treatment of Cancer Quality-of-Life Questionnaire-C30 [48]. LLOQ: lower limit of quantification.

### Genotyping

SNP selection was based on consistency with prior EPOS analyses [10, 16]. DNA was extracted from EDTA–treated whole blood [10] and genotyped for 20 SNPs in 14 genes involved in innate immune activation [*TLR4* (rs4986790, rs4986791); *TLR2* (rs3804100); *MD2* (rs11466004); *MYD88* (rs6853)], mediating inflammation [*IL1B* (rs1143627, rs1143634, rs16944); *CASP1* (rs554344, rs580253); *IL6* (rs10499563); *IL6R* (rs8192284); *IL10* (rs1800871, rs1800896); *IL2* (rs2069762); *CRP* (rs2794521); *TGFB1* (rs11466314, rs1800469); *TNFA* (rs1800629)], and neuronal adaptation [*BDNF* (rs6265)], as described previously [16–19].

The following SNPs had been genotyped previously [10]: *COMT* (rs4680), *OPRM1* (rs1799971), *ARRB2* (rs3786047, rs1045280, rs2271167, rs2036657), and *ABCB1* (rs1045642, rs2235013, rs1128503, rs4437575, rs2235033, rs1202170, rs7802773). For *ABCB1*, only rs1045642, rs2235013 and rs1128503 were included in the final analysis based on existing evidence of phenotype associations and near complete linkage disequilibrium (LD) (r^2^≥0.9) with the other *ABCB1* SNPs (data not shown).

Further SNP details are provided in Supplementary Table S1.

### Measures of morphine response

Pathological, physiological and genetic variables were examined for their association with four measures of morphine response: “pain control”; “cognitive dysfunction”; “sickness responder”; and “opioid adverse event complaint”.

As previously for fentanyl [16], patients with average pain <4 on an 11-point NRS in the BPI were categorized as having “pain control” [20]. Patients with total MMSE ≤23 were categorised as having “cognitive dysfunction” [13, 20]. Patients who reported two or more of the following were classified as “sickness responders”: nausea ≥50 (EORTC QLQ-C30 nausea and vomiting scale); tiredness ≥3 (EORTC QLQ-C30 item “Were you tired?”); and depression ≥3 (EORTC QLQ-C30 item “Did you feel depressed?” (“Not at all” or “A little” versus “Quite a bit” or “Very much”). Based on our [16] and other previous EPOS publications [20, 21], patients were categorised as “opioid adverse event complaint” if they reported nausea ≥50 (EORTC QLQ-C30 nausea and vomiting scale); constipation ≥50 (EORTC QLQ-C30 constipation scale); tiredness ≥3 (EORTC QLQ-C30 item “Were you tired?”); and/or had a total MMSE of 23 or less (“cognitive dysfunction”).

### Data analysis

Data were analysed in R [22] unless indicated otherwise. Chi-square analysis was used to test for genotype deviations from Hardy-Weinberg Equilibrium. The co-incidence of specific adverse events (nausea, tiredness, constipation, depression, cognitive dysfunction) was investigated using Fishers Exact Test (fisher.test function of the R base package [22]).

Distributions of continuous variables were assessed using histograms and quantile-quantile (Q-Q) plots, and statistical outliers checked using Grubbs and Rosner tests (grubbs.test function of outliers package [23] and rosnerTest function of EnvStats package [24]). Optimal transformations to normalise the distributions were identified using the boxcox function in the MASS package [25] as required. Transformed (as specified in Table 1) data were then used in all subsequent analyses.

#### Standardised serum morphine and glucuronide concentrations

Because of varying intervals between morphine dose and blood sampling (time-to-sample) (see Table 1), serum morphine, M3G and M6G concentrations were standardised to a set time-to-sample of 660 minutes as described in Supplementary Methods. This standardised estimate of morphine and glucuronides’ concentrations was used in subsequent analyses after appropriate transformations (specified in Table 1), and apart from Table 1, all serum morphine or glucuronides’ concentrations referred to hereafter are the standardised and transformed concentrations unless specified otherwise.

Significant differences in serum morphine, M3G and M6G concentrations between patients with and without each of the four measures of morphine response were assessed by *t*-tests. A sub-analysis of concentration differences between outcomes was also conducted within patients (*n* = 259) with time-to-sample between 9 and 12 h. Correlations (Pearson) between serum morphine, M3G and M6G concentrations were also examined [22].

#### Identification of response predictors

The absence of significant associations between responses and ancestral subgroup was first confirmed by chi-square analysis (*P* > 0.05) before proceeding with further analyses.

Details of the subsequent statistical analysis pipeline are provided in Supplementary Methods. Briefly, major non-genetic variables (listed in Table 1), including serum morphine and glucuronides’ concentrations, to be controlled for in subsequent genotype analyses were identified by LASSO regression.

A step-down regression model selection procedure was used to identify genetic factors associated with different responses, fixing non-genetic predictors identified by LASSO regression as the base model (with first-order interactions with any serum morphine, M3G or M6G concentrations included). Sensitivity analyses were conducted excluding patients with time on opioids of less than 3 days (*n* = 6, all with time on opioids of only 1 day).

Epistasis was also investigated by generalised multifactor dimensionality reduction (GMDR) analysis, incorporating major non-genetic predictors into the response score.

The likelihood of observed model performance (cross-validation error, CVE) occurring by chance within the data for each outcome measure was investigated by comparing model performance against control models using permutations of paired response and non-genetic variable data randomised against paired genetic data. The discriminatory potential of non-genetic and final models was assessed using area under the receiver operating characteristic curve (AUROC). Reported *P*-values have not been adjusted for multiple testing.

## Results

### Genetic variability

Four hundred and thirty-five patients had complete genotype data, with 71 patients missing data for one or more SNPs; allele and genotype frequencies are shown in Supplementary Table S1. No genotype frequencies significantly deviated from Hardy-Weinberg Equilibrium (*P* > 0.2). SNP linkage disequilibrium and haplotypes are detailed in Supplementary Results. There was no significant association between ancestral subgroups and any response (*P* > 0.05).

### Pain control

Of 486 patients with BPI scores, 271 (56%) were classified as having pain control. Serum M3G concentrations were lower in patients with pain control versus without pain control (untransformed median (interquartile range) = 1.0 (0.5–1.9) μM versus 1.2 (0.7–2.3) μM, *P* = 0.006 (*t*-test on transformed data)), as were serum M6G (187 (96–387) nM versus 235 (122–438) nM, *P* = 0.02) and morphine (not significantly) (27 (12–64) nM versus 34 (17–61) nM, *P* = 0.06) concentrations (visualised in Supplementary Figure S2). Similar results were observed in sub-analysis of patients with time-to-sample between 9–12 h (see Supplementary Results).

Serum morphine and M3G (Pearson r = 0.83), morphine and M6G (r = 0.82), and M3G and M6G (r = 0.98) concentrations were all significantly positively correlated (*P* < 2.2 × 10^−^^16^). LASSO regression identified longer time on morphine, prostate cancer and visceral pain as associated with increased pain control, and higher serum M3G concentrations, back pain and depression as associated with reduced pain control, with a slight-modest predictive value (AUROC = 0.66) (Supplementary Table S2). *TLR2* rs3804100 variant carriers were associated with increased pain control, and an interaction between *CASP1* rs554344 and serum M3G concentrations was also identified (rs554344 homozygous variant genotype increased the magnitude of M3G concentration effect). However, cross-validation performance of this model (optimal k = 3.2, CVE = 0.228 < base model = 0.231) was no better than randomised controls [median (25–75^th^ percentile) CVE = 0.228 (0.225–0.230)]. Reflecting this, no genetic regressors were significant predictors of pain control based on nested model comparison (likelihood ratio Chi-square) (*P* > 0.06) (Supplementary Table S2), and there was only a slight improvement in predictive performance of the model with the addition of these regressors (AUROC = 0.70).

### Adverse events

Analysis of the co-incidence of specific adverse events showed cognitive dysfunction was unrelated to other adverse events, but there were significant positive associations between nausea, tiredness, depression and constipation (Table 2).

**Table 2:** Co-incidence of adverse events reported by cancer pain patients receiving slow-release oral morphine.

OR (95% CI)	Nausea	Tiredness	Depression	Constipation
Tiredness	3.2 (1.7–6.2)**** (*n* = 479)			
Depression	2.2 (1.3–3.6)** (*n* = 481)	4.2 (2.5–7.6)**** (*n* = 475)		
Constipation	2.4 (1.5–3.9)*** (*n* = 484)	2.2 (1.5–3.4)*** (*n* = 478)	1.4 (0.95–2.2) (*n* = 480)	
Cognitive Dysfunction	0.96 (0.42–2.0) (*n* = 456)	0.86 (0.46–1.6) (*n* = 450)	1.2 (0.63–2.3) (*n* = 452)	1.0 (0.56–1.9) (*n* = 456)

**P < 0.01, ***P < 0.001, ****P < 0.0001 Fisher’s exact test. *OR* Odds Ratio, *CI* Confidence Interval.

#### Cognitive dysfunction

Of 472 patients with MMSE data, 62 (13%) had cognitive dysfunction. Serum morphine concentrations were significantly higher in patients with cognitive dysfunction (untransformed median (interquartile range) = 40 (20–81) nM) versus without cognitive dysfunction (29 (14–60) nM; *P* = 0.02 (*t*-test on transformed data)). Neither M3G (*P* = 0.3) nor M6G (*P* = 0.2) concentrations were significantly different in patients with cognitive dysfunction (visualised in Supplementary Figure S2). In sub-analysis of patients with time-to-sample between 9 and 12 h, serum morphine (*P* = 0.005), M3G (*P* = 0.01) and M6G (*P* = 0.01) concentrations were all higher in patients with cognitive dysfunction (see Supplementary Results).

In addition to serum morphine concentrations, older age and lower Karnofsky functional status were associated with increased cognitive dysfunction, with a modest predictive value (AUROC = 0.74) (Supplementary Table S3). *IL1B* rs1143627 variant carrier genotype was associated with increased cognitive dysfunction (Supplementary Table S3), however cross-validation performance of this model (optimal k = 4, CVE = 0.106 < base model = 0.107) was no better than randomised controls [median (25–75th percentile) CVE = 0.107 (0.106–0.107)] and had only slightly improved predictive value (AUROC = 0.75). The incidence of cognitive dysfunction in *MYD88* rs6853 carriers (13/108 = 12%) was not significantly lower than wild-type (47/352 = 13%) [OR (95% CI) = 0.89 (0.46–1.7), *P* = 0.9], nor was *MYD88* rs6853 carrier status a significant predictor of cognitive dysfunction after adjusting for serum morphine concentration, age, Karnofsky score and *IL1B* rs1143627 genotype (*P* = 0.8).

#### Sickness response

Of 475 patients with EORTC data, 154 (32%) were classified as “sickness responders”. Serum morphine, M3G and M6G concentrations were not significantly different in “sickness responders” (*P* > 0.4) (visualised in Supplementary Figure S2) (similarly in sub-analysis of patients with time-to-sample between 9 and 12 h; see Supplementary Results). NSAID administration and female sex were associated with increased, and United Kingdom, Iceland or Italian treatment centre was associated with decreased sickness response with modest predictive value (AUROC = 0.73).

*IL2* rs2069762 (co-dominant), *BDNF* rs6265 (variant recessive), *IL6R* rs8192284 (variant dominant), *COMT* rs4680 (co-dominant), *OPRM1* rs1799971 (variant dominant) and *TLR4* rs4986790 (co-dominant) polymorphisms were associated with decreased, and the *IL6* rs10499563 (variant recessive) polymorphism associated with increased, sickness response (Table 3) (optimal k = 2). Adding these genetic factors improved the predictive ability over the non-genetic model (AUROC = 0.80), and the cross-validation performance of this model (CVE = 0.175 versus 0.191 for non-genetic model) was better than any randomised controls [median (25–75th percentile); range CVE = 0.190 (0.189–0.191); 0.183–0.194]. These polymorphisms also demonstrated similar associations with sickness response without accounting for non-genetic factors (data not shown). Univariate analyses of associations between these polymorphisms and each specific symptom (nausea, tiredness, depression) are provided in Supplementary Table S4 and there was less likelihood of nausea in carriers of *OPRM1* rs1799971 (OR = 0.43 (0.23–0.78), *P* = 0.004).

**Table 3:** Variables associated with sickness response in cancer pain patients (n = 456) receiving slow release oral morphine.

Regressor	Adjusted Odds Ratio^a^ (95% CI)		Nested model *P*-value^b^	Relative risk^a^ (95% CI)
Iceland treatment centre^c^	0.18	(0.09–0.34)	3 × 10^-8^	0.33 (0.20–0.54)
UK treatment centre^d^	0.15	(0.05–0.37)	6 × 10^-6^	0.26 (0.11–0.58)
Italy treatment centre^e^	0.08	(0.01–0.29)	2 × 10^-5^	0.17 (0.04–0.69)
Female sex^f^	2.2	(1.4–3.5)	5 × 10^-4^	1.5 (1.2–1.9)
NSAID co-administration^g^	2.0	(1.2–3.3)	0.006	1.4 (1.1–1.8)
*IL2* rs2069762^h^			0.003	
T/G	0.78	(0.49–1.2)		0.88 (0.69–1.1)
G/G	0.20	(0.06–0.52)^**†^		0.35 (0.15–0.82)
*BDNF* rs6265 A/A^i^	0.15	(0.02–0.63)	0.007	0.29 (0.07–1.2)
*IL6R* rs8192284 carrier^h,j^	0.55	(0.34–0.90)	0.02	0.74 (0.57–0.94)
*IL6* rs10499563 C/C^i^	3.3	(1.2–9.3)	0.02	1.8 (1.1–3.0)
*COMT* rs4680^h^			0.08	
G/A	0.60	(0.34–1.1)		0.77 (0.58–1.0)
A/A	0.48	(0.25–0.92)		0.69 (0.50–1.0)
*TLR4* rs4986790 carrier^h,j^	0.49	(0.20–1.1)	0.09	0.66 (0.40–1.1)
*OPRM1* rs1799971 carrier^h,j^	0.64	(0.37–1.1)	0.1	0.79 (0.57–1.1)

^a^Odds Ratio and Relative Risk controlling for all other regressors. Odds ratio and relative risk greater than 1 indicates an association with increased likelihood of sickness response. ^b^Likelihood ratio chi-square test *P*-value testing each term after all others (i.e., nested model comparisons) according to the marginality principle [49]. Reference groups are ^c^non-Iceland treatment centre, ^d^non-UK treatment centre, ^e^non-Italy treatment centre, ^f^male sex, ^g^no NSAID co-administration, ^h^homozygous wildtype genotype, ^i^homozygous wildtype or heterozygous genotype. ^j^Carrier: heterozygous or homozygous variant. ^**^Tukey post-hoc P < 0.01 versus homozygous wild-type. ^†^Tukey post-hoc P < 0.05 versus heterozygous.

#### Opioid adverse event complaint

Of 449 patients with adverse event data, 365 (81%) were classified as opioid “adverse event complainers”. Serum morphine, M3G and M6G concentrations were not significantly different in “adverse event complainers” (*P* > 0.1) (visualised in Supplementary Figure S2) (similarly in sub-analysis of patients with time-to-sample between 9 and 12 h; see Supplementary Results). Denmark and Iceland treatment centres were associated with lower complaint, but with limited predictive value (AUROC = 0.61). *TLR2* rs3804100 carriers were associated with decreased, and *CASP1* rs554344 homozygous variants associated with increased, complaint (Supplementary Table S5) (optimal k = 4). Adding these genetic factors improved the predictive ability over the non-genetic model (AUROC = 0.66), but the cross-validation performance of this model (CVE = 0.146 versus 0.148 for non-genetic model) was within the 15^th^ percentile of randomised controls [median (25–75th percentile) CVE = 0.148 (0.146–0.148)].

An increased penalty (k = 4.1–6.4) reduced the model to *TLR2* rs3804100 as the sole genetic factor (Table 4). Whilst this slightly increased the model CVE (0.1463 versus 0.1458 for model also including *CASP1* rs554344), this was within the 5^th^ percentile of randomised controls at the higher penalty (k = 6.4) [median (25–75th percentile) CVE = 0.148 (0.148–0.148)], with an AUROC of 0.65.

**Table 4:** Variables associated with opioid adverse event complaint in cancer pain patients (n = 438) receiving slow release oral morphine.

Regressor	Adjusted Odds Ratio^a^ (95% CI)		Nested model *P*-value^b^	Relative risk^a^ (95% CI)
Iceland treatment centre^c^	0.38	(0.22–0.67)	0.001	0.82 (0.71–0.94)
Denmark treatment centre^d^	0.13	(0.04–0.45)	0.002	0.53 (0.28–1.0)
*TLR2* rs3804100 carrier^e,f^	0.42	(0.22–0.82)	0.01	0.83 (0.70–1.0)

^a^Odds Ratio and Relative Risk controlling for all other regressors. Odds ratio and relative risk less than 1 indicates an association with decreased likelihood of sickness response. ^b^Likelihood ratio chi-square test *P*-value testing each term after all others (i.e. nested model comparisons) according to the marginality principle [49]. Reference groups are ^c^non-Iceland treatment centre, ^d^non-Denmark treatment centre and ^e^homozygous wildtype genotype. ^f^Carrier: heterozygous or homozygous variant.

No epistatic models for any response measure performed better than randomised dataset controls. Excluding patients receiving opioids for only one day had negligible impact on model adjusted Odds Ratios and *P*-values for pain control, cognitive dysfunction, sickness response and opioid adverse event complaint (Supplementary Tables S6–S10).

## Discussion

**Fig. 1:**
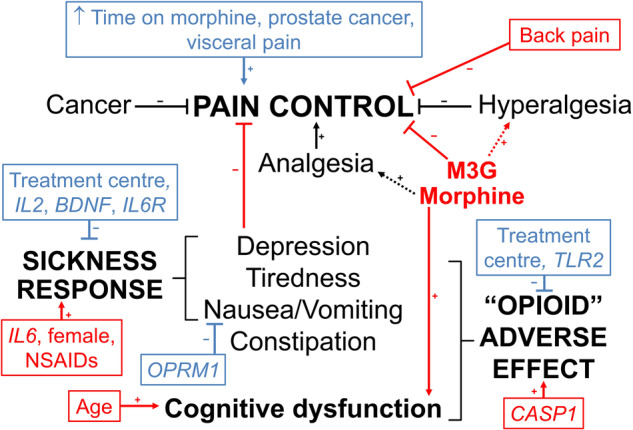
Summary of pharmacogenomic (innate immune, neuronal, neurotrophic) and pharmacokinetic (plasma morphine and -3 and 6-glucuronides), patient and treatment centre factors contributing to pain control and adverse effects in cancer patients receiving sustained-release morphine. + refers to a positive contribution; - refers to negative contribution.

Figure 1 summarises the pharmacogenetic, pharmacokinetic and demographic factors contributing to pain control and adverse effects in these cancer patients receiving sustained-release morphine. A major pharmacokinetic finding was that serum M3G concentrations were significantly lower (median 20%) in patients with pain control. As a pain score of greater than 3 is considered as “unacceptable pain”, its association with higher serum M3G concentrations is clinically significant. This finding has mechanistic plausibility as M3G binds to MD2 the accessory TLR4 protein causing a proinflammatory response [2] and M3G induces hyperalgesia through the MD2/TLR4 complex in rats through changes in the functioning of voltage-gated sodium channels [26]. That M6G was also associated with “unacceptable pain”, although statistically weaker (*P* = 0.02) may simply be due to its hepatic formation from morphine and its renal elimination being mechanistically similar to that of M3G. As morphine, M3G and M6G are all highly significantly positively correlated with each other, any relationship of one of these analytes to an outcome will hold generally for the others. Importantly, morphine concentrations were also higher (although non-significantly) in those with “unacceptable pain”, and therefore any increased rate of metabolism of morphine to M3G in the high serum M3G patients (which would lead to lower morphine concentrations) does not explain the M3G link to “unacceptable pain”. Finally, that M3G showed the most significant relationship of the three analytes to “unacceptable pain”, and was the only analyte retained in LASSO regression, suggests it is the major causative pharmacokinetic variable.

The non-genetic variables of back pain and depression being associated with reduced pain control on opioids is not surprising, especially given the co-occurrence between depression and chronic pain [27]. Our findings do not support that, after accounting for key non-genetic variables like depression and back pain, genotyping for the SNPs investigated would improve predictions of cancer pain attenuation with morphine.

Cognitive dysfunction is a major long term adverse effect of opioids and occurred in 13% of this cohort, similar (18%) to that found with fentanyl [16]. That higher serum morphine, more so than M3G and M6G, was found in those with cognitive dysfunction (median 38% higher) suggests that the metabolites per se are not the major causative contributors but morphine itself, or a combination of morphine and metabolites. This association with serum morphine, using MMSE as the measure, has not been found previously [20] and is likely to reflect differences in study design, analysis and patient cohorts. In keeping with our fentanyl study [16], there was no statistically significant coincidence between cognitive dysfunction and nausea, tiredness, depression or constipation. Apart from serum morphine, that older age and lower Karnofsky score were associated (the latter two modestly) with cognitive dysfunction was also found for EPOS patients treated with other opioids [16, 21], suggesting either an opioid-class effect or opioid-independent relationships between age, Karnofsky and MMSE scores [21, 28]. However, in contrast to our previous findings for transdermal fentanyl [16], the incidence of cognitive dysfunction was not associated with *MYD88* (adaptor protein involved in innate immune cell signalling via Toll-like receptors, which can be activated by opioids [2]) rs6853 genotype, suggesting that the role of *MYD88* genotype in fentanyl cognitive dysfunction is not an opioid-class effect.

Sickness response was based on patients reporting two of nausea, tiredness and depression and were all positively and significantly associated with each other. Three treatment centres (Iceland, UK, Italy) had less sickness responders than the others. Whether this reflects a different therapeutic approach to treatment or their cohort is phenotypically or demographically different is unknown but requires investigation. That more females were sickness responders than males supports the now well-recognised finding that females experience more adverse drug effects than males [29], likely a reflection of innate and adaptive immune response differences [30], as we also found for fentanyl [16]. Finally, NSAID use association with sickness response is a new finding, the mechanisms are unknown but requires further assessment, especially the recent finding of NSAID use being associated with pain persistence in those with low back pain [31]. That nausea was less likely in carriers of *OPRM1* rs1799971 is consistent with the reduced expression/function of the variant [32]. In contrast to fentanyl, three gene SNPs for *IL2* (homozygous variant), *BDNF* (homozygous variant) and *IL6R* (carriers) were associated with decreased sickness response. The *IL2* variant leads to increased secretion of the cytokine and hence proinflammation and has been associated with increased postoperative pain and morphine use [18] and the *BDNF* variant causes reduced activity-dependent BDNF secretion [33, 34] and is associated with increased depression symptoms in cancer patients [35–37], the opposite to that found here. In contrast, *IL6* homozygous rs10499563 variant (C/C) genotype was associated with increased sickness response and is difficult to explain mechanistically as it is associated with lower IL6 expression and serum concentrations and so would be predicted to be less proinflammatory [38].

For opioid complainers, there were significantly fewer complainers in the *TLR2* variant carrier group. In mice, morphine induces microglial expression of TLR2 which plays an essential role in morphine-induced microglial activation and increased IL6 expression [39, 40]; the variant is likely to blunt [41, 42] this CNS proinflammatory effect leading to less complainers of the combination of significant nausea, constipation, tiredness and cognitive dysfunction.

Though analysed independently, there is significant overlap in symptoms defining sickness response and opioid adverse event. These symptoms, along with their genetic interactions, could reflect a combination of the underlying disease per se, morphine use and/or their interactions (including negative feedback mechanisms) which might be further explored through Bayesian networks or related network/pathway-based approaches [43, 44].

Relevant to all assessed outcomes are the very wide ranges of time on opioids (1-3725 days) and morphine doses (10–1600 mg/day), and variability in time-to-sample for serum concentration analyses, within this cohort. Whilst the latter was addressed by standardising concentrations to a set time-to-sample, and sub-analyses within a narrower range of time-to-sample confirmed major serum concentration-response findings, this remains a limitation of the study in characterising these relationships. Reducing heterogeneity in time on opioids and morphine doses could potentially improve the ability to detect statistically significant genotype differences in outcomes. However, this variability reflects (and continues to be relevant to) the diversity of the cancer pain population, with no statistical outliers for these variables (excepting six patients with only one day of opioid treatment, the exclusion of whom had negligible impact on results). Through prudent use of existing data and samples, the candidate gene/pathway and analysis approaches employed reflect the available sample size and expectation of modest effect sizes for multiple SNPs (versus large effects of single SNPs), respectively, where a standard GWAS approach would lack sensitivity. The inclusion of specific SNPs was consistent with prior EPOS analyses [10, 16] for which SNPs were selected based on knowledge of candidate genes contributing to opioid neuroimmunopharmacology pathways, and published associations between their SNPs and relevant phenotypes, at the time. This study adds important data on the polymorphisms examined, but is not exhaustive, and other genetic predictors (including immune and neuronal polymorphisms (e.g. [45]) not covered in the targeted panel employed) may help further explain interpatient variability in these outcomes.

In summary, innate immune, neuronal and neurotrophic genetics contributed to opioid adverse effects, but not pain intensity which is influenced by morphine-3-glucuronide, in cancer patients receiving morphine.

### Supplementary information

Supplementary Methods

Supplementary Results

Supplementary Tables

Supplementary Figures

## Data Availability

These data are made available to researchers on request to the European Palliative Care Research Network which is responsible for the European Pharmacogenetic Opioid Study coordinated through St. Olav´s University Hospital, Trondheim University, Norway (contact: pal.klepstad@ntnu.no).
