# Supplementary material for: Pharmacokinetic and neuroimmune pharmacogenetic impacts on slow-release morphine cancer pain control and adverse effects
Source: Pharmacogenomics J. 2024 Jun 1;24(3):18. doi: 10.1038/s41397-024-00339-w (PMC11144121; doi:10.1038/s41397-024-00339-w)
Supplement: Supplementary file 1 — Supplementary Methods [file 41397_2024_339_MOESM1_ESM.pdf]

Supplementary Methods

This file contains Supplementary Methods (including references) outlining the details of the statistical analysis pipeline for the manuscript entitled "Pharmacokinetic and neuroimmune pharmacogenetic impacts on slow-release morphine cancer pain control and adverse effects."

<u>Standardised serum morphine and glucuronide concentrations</u>

Because of varying intervals between morphine dose and blood sampling (time-to-sample) (see manuscript Table 1), serum morphine, M3G and M6G concentrations were standardised to a set time-to-sample as follows: of 543 Caucasian participants receiving slow-release oral morphine, 504 had time-to-sample data, from which a further 2 participants were excluded from the following calculations due to time-to-sample greater than 1000 minutes. Of the remaining 502 participants, all had quantitative M3G (>35 nmol/L) and M6G (>2 nmol/L) concentration data, whilst 20 participants had serum morphine concentrations below the lower limit of quantification (3.5 nmol/L [1]) for which standardised concentrations could not be estimated. Best transformations for linear relationships between serum morphine concentrations, dose and time-to-sample were λ = -0.1 box-cox transformation, log-transformation, and squared-transformation, respectively. Best transformations for linear relationships between serum M3G or M6G concentrations, dose and time-to-sample were log-transformation, log-transformation, and squared-transformation, respectively.

Linear regression was used to determine the scheduled morphine dose and time-to-sample versus serum concentration relationships, from which individual serum concentrations were standardised to a 660-minute time-to-sample concentration.

This standardised estimate of morphine and glucuronides' concentrations exposure was used in subsequent analyses after appropriate transformations (specified in manuscript Table 1), and apart from Table 1, all serum morphine or glucuronides' concentrations referred to in the manuscript are the standardised and transformed concentrations unless specified otherwise.

Ancestral and non-genetic variables

Associations between each measure of morphine response and ancestral subgroup were checked by chi-square test.

Differences in serum morphine, M3G and M6G concentrations were first compared between outcome groups using two-tailed unpaired t-tests. Following this preliminary analysis, major non-genetic variables (listed in manuscript Table 1) to be controlled for in subsequent genotype analyses were then identified by LASSO regression (generalised binomial) using cross-validation to identify the appropriate lambda penalty (largest lambda within 1 standard error of the minimum cross-validated error (CVE)) (glmnet and cv.glmnet functions in glmnet package [2]). When the preliminary (t-test) analyses were significant for either serum morphine, M3G or M6G concentrations, a half (0.5) penalty was applied to that respective concentration variable for the LASSO regression. Otherwise, the same penalty was applied to serum concentrations as to other non-genetic variables.

Co-medications that showed positive associations with an outcome for which they were indicated (e.g. breakthrough opioids for pain relief, or antiemetics for nausea and vomiting) were excluded from analysis. However, where they showed negative associations, they were retained in order to account for their mitigating effects.

If confirmed to be significant ($P < 0.05$) by nested model comparison (Wald chi-square), non-genetic variables selected from LASSO regression were then used as a base model for subsequent analyses of genetic polymorphisms.

Candidate polymorphism analyses

Only SNPs with minor allele frequencies greater than 2.5% were included in analyses. Linkage disequilibrium (LD) between SNPs within *IL1B*, *IL10*, *TLR4*, *CASP1* and *ARRB2* was determined using the LD function of the "genetics" package [3]. Where feasible, tightly linked SNPs were combined into haplotypes before further analysis.

Based on the expectation of modest effect sizes for multiple SNPs, rather than large effects of single SNPs, a step-down regression model selection procedure based on Akaike Information Criterion (AIC) (stepAIC function in MASS package [4]) was used to identify genetic factors associated with different responses. Fixing non-genetic predictors as the base model, leave-one-out cross-validation was used to identify the optimal k penalty ($k \geq 2$) to minimise CVE (cv.glm function in boot package [5,6]). If a genetic factor was included at the optimal k (initially using a co-dominance model), different dominance models (wildtype dominant and variant dominant) were tested for model improvement (reduced CVE). If a wildtype or variant dominant model was chosen for a genetic factor, the k optimisation procedure was repeated, and so on until a stable best model was achieved.

Given the number of genetic factors investigated, the likelihood of chance associations within the data for each outcome measure was investigated by comparing the CVE of the final model to the CVE of models generated using 100-200 permutations of the response (paired to any non-genetic variables) randomised against paired genetic data.

Given small proportions of missing data for many variables, a pairwise deletion approach was taken to make full use of the available data for each series of analysis; Supplementary Figure S1 provides a flowchart outlining the exclusion and inclusion criteria and consequent sample sizes for each analysis.

Possible epistatic interactions were investigated by generalised multifactor dimensionality reduction analysis (Generalised Multifactor Dimensionality Reduction v0.9 [7,8]). Phenotype scores were generated within the software using the response measure and any non-genetic variables identified from LASSO regression. Two-, 3-, and 4-way gene interactions were investigated using 5-fold cross-validation for 10 different random seeds. Candidate models within each level were selected based on the following criteria: the highest cross-validation consistency (>20%); median testing balanced accuracy greater than the median + 1 standard deviation testing balanced accuracy of unselected models; and median testing balanced accuracy greater than the median + 1 standard deviation of the highest cross-validation consistency models generated from 10 randomised response data sets.

Receiver operating characteristic (ROC) curves of non-genetic and final models were generated using the “ROC” function of the “epicalc” package [9]. Likelihood ratio chi-square test P-values were obtained using the “Anova” function of the “car” package [10], testing each term after all others (i.e. nested model comparisons) according to the marginality principle. Estimates (and 95% confidence intervals) of relative risk were obtained using the “vcovHC” function of the “sandwich” package [11,12].

Supplementary Methods References

1. Fladvad, T, Klepstad, P, Langaas, M, Dale, O, Kaasa, S, Caraceni, A, *et al.* Variability in UDP-glucuronosyltransferase genes and morphine metabolism: observations from a cross-sectional multicenter study in advanced cancer patients with pain. Pharmacogenet Genomics. 2013;23(3):117-126.
2. Friedman, J, Hastie, T, Tibshirani, R. Regularization Paths for Generalized Linear Models via Coordinate Descent. Journal of Statistical Software. 2010;33(1):1-22.
3. Warnes G with contributions from Gregor Gorjanc; Friedrich Leisch and Michael Man. genetics: Population Genetics. R package version 1.3.8.1. http://CRAN.R-project.org/package=genetics. 2013.
4. Venables, W, Ripley, B. Modern Applied Statistics with S. 4th ed. New York: Springer; 2002.
5. Angelo Canty and Brian Ripley. boot: Bootstrap R (S-Plus) Functions. R package version 1.3-9. 2013.
6. Davison, AC, Hinkley, DV. Bootstrap Methods and Their Applications. Cambridge: Cambridge University Press; 1997.
7. Chen, GB, Xu, Y, Xu, HM, Li, MD, Zhu, J, Lou, XY. Practical and theoretical considerations in study design for detecting gene-gene interactions using MDR and GMDR approaches. PLoS One. 2011;6(2):e16981.
8. Lou, XY, Chen, GB, Yan, L, Ma, JZ, Zhu, J, Elston, RC, *et al.* A generalized combinatorial approach for detecting gene-by-gene and gene-by-environment interactions with application to nicotine dependence. Am J Hum Genet. 2007;80(6):1125-1137.
9. Virasakdi Chongsuvivatwong. epicalc: Epidemiological calculator. R package version 2.15.1.0. http://CRAN.R-project.org/package=epicalc. 2012.

10. Fox, J, Weisberg, S. An {R} Companion to Applied Regression, Second Edition. Thousand Oaks, CA: Sage; 2011.

11. Zeileis, A. Econometric Computing with HC and HAC Covariance Matrix Estimators. Journal of Statistical Software. 2004;11(10):1-17.

12. Zeileis, A. Object-Oriented Computation of Sandwich Estimators. Journal of Statistical Software. 2006;16(9):1-16.
