# Supplementary material for: Pharmacokinetic and neuroimmune pharmacogenetic impacts on slow-release morphine cancer pain control and adverse effects
Source: Pharmacogenomics J. 2024 Jun 1;24(3):18. doi: 10.1038/s41397-024-00339-w (PMC11144121; doi:10.1038/s41397-024-00339-w)
Supplement: Supplementary file 2 — Supplementary Results [file 41397_2024_339_MOESM2_ESM.pdf]

**Supplementary Results**

This file contains Supplementary Results for the manuscript entitled “Pharmacokinetic and neuroimmune pharmacogenetic impacts on slow-release morphine cancer pain control and adverse effects”, relating to linkage disequilibrium and haplotypes of the SNPs investigated, and sub-analysis of concentration differences between outcomes.

*Linkage disequilibrium and haplotypes*

*IL1B* rs16944 and rs1143627 were in near complete LD (one patient rs16944 C/C and rs1143627 T/C. D’ = 0.9998, $r^2$ = 0.995), therefore only rs1143627 was included in further analyses. *IL1B* rs1143627 and rs1143634 were in significant but incomplete linkage disequilibrium (D’ = 0.79, $r^2$ = 0.09, $\chi^2$ P < 2 x $10^{-16}$), and were analysed separately. *CASP1* rs554344 and rs580253 were in near complete LD (one patient rs554344 C/C and rs580253 G/A. D’ = 0.9997, $r^2$ = 0.993), therefore only rs554344 was included in further analyses. *TLR4* rs4986790 and rs4986791 were in near complete LD strong LD (one patient rs4986790 A/G and rs4986791 C/C, and one patient rs4986790 A/A and rs4986791 C/T. D’ = 0.98, $r^2$ = 0.95), therefore only rs4986790 was included in further analyses.

*ARRB2* rs3786047, rs1045280, rs2271167 and rs2036657 were all in strong LD (D’ > 0.97; $r^2$ > 0.92) and could be categorised into 3 diplotypes: “Wildtype” (all wildtype genotype, n = 53), “heterozygous” (all heterozygous genotype, n = 204; or at least 2 SNPs heterozygous and remaining wildtype or variant, n = 11), “variant” (all variant genotype, n = 218; or heterozygous for 1 SNP and remaining variant, n = 7).

*Sub-analysis of concentration differences between outcomes in patients with time-to-sample between 9-12 hours*

Within patients with time-to-sample between 9-12 hours, serum M3G concentrations were significantly lower in patients with pain control (untransformed median (interquartile range) = 0.9 (0.5-1.8) µM (n=139) versus 1.2 (0.6-2.3) µM (n=116), P=0.03 (t-test on transformed data)). Serum M6G (181 (88-382) nM (n=139) versus 243 (115-465) nM (n=116), P=0.09) and morphine (24 (11-62) nM (n=130) versus 35 (15-60) nM (n=113), P=0.07) concentrations were non-significantly lower in patients with pain control versus without pain control, respectively.

Within patients with time-to-sample between 9-12 hours, serum morphine concentrations were significantly higher in patients with cognitive dysfunction (untransformed median (interquartile range) = 57 (22-128) nM, n=30) versus without cognitive dysfunction (28 (13-52) nM, n=193; t-test P=0.005 (t-test on transformed data), as were M3G concentrations (1.8 (0.8-3.0) µM (n=31) versus 1.1 (0.5-2.1) µM (n=203), P=0.01) and M6G concentrations (321 (161-803) nM (n=31) versus 196 (99-405) nM (n=203), P=0.01.

Within patients with time-to-sample between 9-12 hours, serum morphine, M3G and M6G concentrations were not significantly different in "sickness responders" (P>0.1) nor "adverse event complainers" (P>0.7).
