# Supplementary material for: Pharmacokinetic and neuroimmune pharmacogenetic impacts on slow-release morphine cancer pain control and adverse effects
Source: Pharmacogenomics J. 2024 Jun 1;24(3):18. doi: 10.1038/s41397-024-00339-w (PMC11144121; doi:10.1038/s41397-024-00339-w)
Supplement: Supplementary file 3 — Supplementary Tables [file 41397_2024_339_MOESM3_ESM.pdf]

Supplementary Tables

This file contains Supplementary Tables S1-S11 for the manuscript entitled "Pharmacokinetic and neuroimmune pharmacogenetic impacts on slow-release morphine cancer pain control and adverse effects".

Supplementary Table S1. SNP allele and genotype frequencies in EPOS cancer pain patients receiving slow-release oral morphine.

**Gene**	**SNP**	**Common Name**	**Location**	**Nucleotide change**	**Amino acid change**	**Variant allele frequency**	**Genotype n (frequency)**		
							**Wt/Wt**	**Wt/V**	**V/V**
IL6	rs10499563	(-6331T>C)	5'	T>C		0.19	327 (0.66)	146 (0.29)	22 (0.04)
IL1B	rs1143627	(-31T>C)	5'	T>C		0.33	218 (0.44)	223 (0.45)	54 (0.11)
IL1B	rs16944	(-511C>T)	5'	C>T		0.33	219 (0.44)	222 (0.45)	54 (0.11)
IL1B	rs1143634	(3954C>T)	exon 5	C>T	synonymous	0.23	296 (0.60)	168 (0.34)	30 (0.06)
LY96	rs11466004		exon 5	C>T	Ser157Pro	0.02	473 (0.96)	18 (0.04)	1 (0.00)
OPRM1	rs1799971	(118A>G)	exon 1	A>G	Asn40Asp[a]	0.14	369 (0.75)	118 (0.24)	8 (0.02)
TGFB1	rs11466314	(-1287G>A)	5'	G>A		0.00	495 (1.00)	0 (0.00)	0 (0.00)
TGFB1	rs1800469	(-509C>T)	5'	C>T		0.30	246 (0.50)	199 (0.40)	49 (0.10)
TNFa	rs1800629	(-308 G>A)	5'	G>A		0.17	339 (0.68)	139 (0.28)	17 (0.03)
IL10	rs1800871	(-819C>T)	5'	C>T		0.24	288 (0.58)	176 (0.36)	31 (0.06)
IL10	rs1800896	(-1082G>A)	5'	G>A		0.52	121(0.25)	229 (0.46)	143 (0.29)
IL2	rs2069762	(-330T>G)	5'	T>G		0.29	248 (0.50)	203 (0.41)	44 (0.09)
CRP	rs2794521	(-717T>C)	5'	T>C		0.27	257 (0.52)	202 (0.41)	34 (0.07)
TLR2	rs3804100	(1350T>C)	exon 3	T>C	synonymous	0.07	431 (0.87)	62 (0.13)	2 (0.00)
TLR4	rs4986790	(896A>G)	exon 3	A>G	Asp299Gly	0.05	451 (0.91)	43 (0.09)	1 (0.00)
TLR4	rs4986791	(1196C>T)	exon 3	C>T	Thr399Ile	0.04	423 (0.91)	44 (0.09)	0 (0.00)
CASP1	rs554344	(10643G>C)	3' UTR	G>C		0.19	332 (0.67)	136 (0.27)	27 (0.05)
CASP1	rs580253	(5352G>A)	exon 5/6	G>A	synonymous	0.19	331 (0.68)	133 (0.27)	26 (0.05)
BDNF	rs6265	(196G>A)	exon 6	G>A	Val66Met	0.19	326 (0.66)	147 (0.30)	20 (0.04)
MYD88	rs6853		3' UTR	A>G		0.12	381 (0.77)	106 (0.21)	7 (0.01)
IL6R	rs8192284		exon 9	A>C	Asp358Ala	0.41	168 (0.34)	246 (0.50)	81 (0.16)
COMT	rs4680	(472G>A)	exon 4	G>A	Val158Met	0.53	103 (0.21)	255 (0.52)	134 (0.27)
ARRB2	rs3786047		intron	A>G		0.67	53 (0.11)	209 (0.43)	222 (0.46)
ARRB2	rs1045280		exon 11	C>T	Synonymous	0.67	54 (0.11)	210 (0.43)	222 (0.46)
ARRB2	rs2271167		intron	A>G		0.67	53 (0.11)	207 (0.44)	215 (0.45)
ARRB2	rs2036657		3’ UTR	G>A		0.67	54 (0.11)	208 (0.43)	221 (0.46)
ABCB1	rs1045642	(3435C>T)	exon 26	G>A	Synonymous	0.55	91 (0.18)	263 (0.53)	144 (0.29)
ABCB1	rs2235013		intron			0.48	125 (0.26)	246 (0.52)	106 (0.22)
ABCB1	rs1128503	(1236C>T)	exon 12	G>A	Synonymous	0.44	137 (0.29)	261 (0.54)	81 (0.17)

[a]Also known as 335A>G Asn102Asp.; Wt= Wildtype; V=variant

Supplementary Table S2. Variables associated with adequate pain control in cancer pain patients (n = 414) receiving slow-release oral morphine.

Regressor	Adjusted Odds Ratio[a] (95%CI)		Nested model P-value[b]
**Time on opioids (log(days))**	1.3	(1.1 to 1.5)	0.0008
**Standardised serum morphine-3-glucuronide concentration (M3G) (log(µM))**	0.73	(0.58 to 0.90)	0.001
**Depression[c]**	0.49	(0.30 to 0.77)	0.002
**Visceral pain[d]**	2.43	(1.22 to 5.06)	0.01
**Prostate cancer[e]**	2.11	(1.13 to 4.12)	0.02
**Back pain[f]**	0.62	(0.40 to 0.96)	0.03
***TLR2* rs3804100 variant carrier[g,i]**	1.70	(0.90 to 3.30)	0.1
***CASP1* rs554344 homozygous variant[h]**	$1.1x10^4$	(1.12 to $2.4x10^9$)	0.2
**M3G x *CASP1* rs554344 homozygous variant[h]**	0.32	(0.06 to 1.06)	0.06

[a]Odds Ratio controlling for all other regressors. Odds ratio greater than 1 indicates an association with increased likelihood of pain control. [b]Likelihood ratio chi-square test P-value testing each term after all others (i.e. nested model comparisons) according to the marginality principle [Fox, J, Weisberg, S. An R Companion to Applied Regression. Third ed. Thousand Oaks, CA: Sage; 2019]. [c]EORTC QLQ-C30: "Did you feel depressed?" ("Not at all" or "A little" (reference) versus "Quite a bit" or "Very much"). Reference groups are [d]non-visceral pain, [e]non-prostate cancer, [f]non-back pain, [g]homozygous wildtype genotype and [h]homozygous wildtype or heterozygous genotype. [i]Carrier: heterozygous or homozygous variant.

Supplementary Table S3. Variables associated with cognitive dysfunction in cancer pain patients (n = 412) receiving slow-release oral morphine.

**Regressor**	**Adjusted Odds Ratio**[a] (95% CI)		**Nested model P-value**[b]
**Standardised serum morphine concentration (µM)**[c]	1.70	(1.11 to 2.66)	0.02
**Age**^{2}	1.0004	(1.0002 to 1.0006)	0.001
**Karnofsky score**	0.97	(0.95 to 0.99)	0.001
***IL1B* rs1143627 variant carrier**[d]	1.86	(0.99 to 3.62)	0.05

[a]Odds Ratio controlling for all other regressors. Odds ratio greater than 1 indicates an association with increased likelihood of cognitive dysfunction. [b]Likelihood ratio chi-square test P-value testing each term after all others (i.e. nested model comparisons) according to the marginality principle [Fox, J, Weisberg, S. An R Companion to Applied Regression. Third ed. Thousand Oaks, CA: Sage; 2019]. [c]Box-cox λ = -0.1 transformed. [d]Heterozygous or homozygous variant (homozygous wildtype as reference).

Supplementary Table S4. Univariate genotype associations with nausea, tiredness and depression in cancer pain patients receiving slow-release oral morphine.

	Nausea			Tiredness			Depression		
**Regressor**	**Odds Ratio**	(95% CI)	**P-value[a] (n)**	**Odds Ratio**	(95% CI)	**P-value[a] (n)**	**Odds Ratio**	(95% CI)	**P-value[a] (n)**
***IL2* rs2069762[b]**			0.07 (474)			**0.03** (468)			0.2 (470)
T/G	0.79	(0.49 to 1.3)		0.86	(0.57 to 1.3)		0.80	(0.52 to 1.2)	
G/G	0.33	(0.10 to 0.87)		0.41	(0.21 to 0.80)*		0.54	(0.23 to 1.1)	
***BDNF* rs6265 A/A[c]**	0.43	(0.07 to 1.5)	0.2 (472)	0.39	(0.14 to 1.0)	0.05 (466)	0.25	(0.04 to 0.90)	**0.03** (468)
***IL6R* rs8192284 carrier[b,d]**	0.77	(0.48 to 1.2)	0.3 (474)	0.76	(0.50 to 1.1)	0.2 (468)	0.93	(0.61 to 1.4)	0.7 (470)
***IL6* rs10499563 C/C[c]**	1.62	(0.56 to 4.1)	0.3 (474)	1.65	(0.63 to 5.1)	0.3 (468)	0.96	(0.33 to 2.4)	0.9 (470)
***COMT* rs4680[b]**			0.2 (471)			0.9 (465)			0.2 (467)
G/A	0.62	(0.36 to 1.1)		0.91	(0.55 to 1.5)		0.69	(0.42 to 1.1)	
A/A	0.63	(0.34 to 1.2)		0.88	(0.50 to 1.5)		0.64	(0.36 to 1.1)	
***TLR4* rs4986790 carrier[b,d]**	1.04	(0.71 to 1.6)	0.8 (474)	0.96	(0.67 to 1.3)	0.8 (468)	1.07	(0.76 to 1.6)	0.7 (470)
***OPRM1* rs1799971 carrier[b,d]**	0.43	(0.23 to 0.78)	**0.004** (474)	0.74	(0.48 to 1.1)	0.2 (468)	0.76	(0.47 to 1.2)	0.2 (470)

[a]Chi-square test. Reference groups are [b]homozygous wildtype genotype and [c]homozygous wildtype or heterozygous genotype. [d]Carrier: heterozygous or homozygous variant. Odds ratio and relative risk greater than 1 indicates an association with increased likelihood of adverse event. *Tukey post-hoc $P < 0.05$ versus homozygous wild-type.

Supplementary Table S5. Variables associated with opioid adverse event complaint in cancer pain patients (n = 438) receiving slow-release oral morphine (alternative model).

Regressor	Adjusted Odds Ratio[a] (95% CI)		Nested model P-value[b]
**Iceland treatment centre[c]**	0.38	(0.22 to 0.67)	0.001
**Denmark treatment centre[d]**	0.14	(0.04 to 0.48)	0.002
***TLR2* rs3804100 variant carrier[e,g]**	0.43	(0.23 to 0.84)	0.01
***CASP1* rs554344 C/C[f]**	5.08	(1.02 to 92.2)	0.045

[a]Odds Ratio controlling for all other regressors. Odds ratio less than 1 indicates an association with decreased likelihood of sickness response. [b]Likelihood ratio chi-square test P-value testing each term after all others (i.e. nested model comparisons) according to the marginality principle [Fox, J, Weisberg, S. An R Companion to Applied Regression. Third ed. Thousand Oaks, CA: Sage; 2019]. Reference groups are [c]non-Iceland treatment centre, [d]non-Denmark treatment centre, [e]homozygous wildtype genotype and [f]homozygous wildtype or heterozygous genotype. [g]Carrier: heterozygous or homozygous variant.

Supplementary Table S6. Variables associated with adequate pain control in cancer pain patients (n = 409)_receiving slow-release oral morphine, excluding patients receiving opioids for only one day.

**Regressor**	**Adjusted Odds Ratio**[a] (95%CI)		**Nested model P-value**[b]
**Time on opioids (log(days))**	1.3	(1.1 to 1.6)	0.0003
**Standardised serum morphine-3-glucuronide concentration (M3G) (log(µM))**	0.71	(0.56 to 0.88)	0.0005
**Depression**[c]	0.46	(0.29 to 0.73)	0.001
**Visceral pain**[d]	2.37	(1.17 to 5.02)	0.02
**Prostate cancer**[e]	2.10	(1.11 to 4.10)	0.02
**Back pain**[f]	0.58	(0.37 to 0.90)	0.01
***TLR2* rs3804100 variant carrier**[g,i]	1.74	(0.92 to 3.38)	0.09
***CASP1* rs554344 homozygous variant**[h]	4.9×10^4	(3.02 to 3.4×10^{10})	0.3
**M3G x *CASP1* rs554344 homozygous variant**[h]	0.25	(0.04 to 0.91)	0.03

[a]Odds Ratio controlling for all other regressors. Odds ratio greater than 1 indicates an association with increased likelihood of pain control. [b]Likelihood ratio chi-square test P-value testing each term after all others (i.e. nested model comparisons) according to the marginality principle [Fox, J, Weisberg, S. An R Companion to Applied Regression. Third ed. Thousand Oaks, CA: Sage; 2019]. [c]EORTC QLQ-C30: "Did you feel depressed?" ("Not at all" or "A little" (reference) versus "Quite a bit" or "Very much"). Reference groups are [d]non-visceral pain, [e]non-prostate cancer, [f]non-back pain, [g]homozygous wildtype genotype and [h]homozygous wildtype or heterozygous genotype. [i]Carrier: heterozygous or homozygous variant.

Supplementary Table S7. Variables associated with cognitive dysfunction in cancer pain patients (n = 408) receiving slow-release oral morphine, excluding patients receiving opioids for only one day.

Regressor	Adjusted Odds Ratio[a] (95% CI)		Nested model P-value[b]
**Standardised serum morphine concentration (µM)[c]**	1.78	(1.15 to 2.81)	0.009
**Age^2**	1.0004	(1.0001 to 1.0006)	0.002
**Karnofsky score**	0.97	(0.95 to 0.99)	0.0008
***IL1B* rs1143627 variant carrier[d]**	1.82	(0.96 to 3.56)	0.07

[a]Odds Ratio controlling for all other regressors. Odds ratio greater than 1 indicates an association with increased likelihood of cognitive dysfunction. [b]Likelihood ratio chi-square test P-value testing each term after all others (i.e. nested model comparisons) according to the marginality principle [Fox, J, Weisberg, S. An R Companion to Applied Regression. Third ed. Thousand Oaks, CA: Sage; 2019]. [c]Box-cox λ = -0.1 transformed. [d]Heterozygous or homozygous variant (homozygous wildtype as reference).

Supplementary Table S8. Variables associated with sickness response in cancer pain patients (n = 452) receiving slow release oral morphine, excluding patients receiving opioids for only one day.

**Regressor**	**Adjusted Odds Ratio**[a] (95% CI)		**Nested model P-value**[b]
**Iceland treatment centre**[c]	0.18	(0.09 to 0.34)	3×10^{-8}
**UK treatment centre**[d]	0.15	(0.05 to 0.37)	6×10^{-6}
**Italy treatment centre**[e]	0.08	(0.01 to 0.29)	3×10^{-5}
**Female sex**[f]	2.2	(1.4 to 3.4)	7×10^{-4}
**NSAID co-administration**[g]	2.0	(1.2 to 3.3)	0.007
***IL2* rs2069762**[h]			0.004
T/G	0.77	(0.48 to 1.2)	
G/G	0.20	(0.06 to 0.55)[**†]	
***BDNF* rs6265 A/A**[i]	0.15	(0.02 to 0.64)	0.007
***IL6R* rs8192284 carrier**[h,j]	0.55	(0.34 to 0.89)	0.02
***IL6* rs10499563 C/C**[i]	3.3	(1.2 to 9.3)	0.02
***COMT* rs4680**[h]			0.09
G/A	0.58	(0.33 to 1.0)	
A/A	0.50	(0.26 to 0.96)	
***TLR4* rs4986790 carrier**[h,j]	0.50	(0.20 to 1.1)	0.09
***OPRM1* rs1799971 carrier**[h,j]	0.62	(0.36 to 1.1)	0.09

[a]Odds Ratio controlling for all other regressors. Odds ratio greater than 1 indicates an association with increased likelihood of sickness response. [b]Likelihood ratio chi-square test P-value testing each term after all others (i.e. nested model comparisons) according to the marginality principle [Fox, J, Weisberg, S. An R Companion to Applied Regression. Third ed. Thousand Oaks, CA: Sage; 2019]. Reference groups are [c]non-Iceland treatment centre, [d]non-UK treatment centre, [e]non-Italy treatment centre, [f]male sex, [g]no NSAID co-administration, [h]homozygous wildtype genotype, [i]homozygous wildtype or heterozygous genotype. [j]Carrier: heterozygous or homozygous variant. [**]Tukey post-hoc P < 0.01 versus homozygous wild-type. [†]Tukey post-hoc P < 0.05 versus heterozygous.

Supplementary Table S9. Variables associated with opioid adverse event complaint in cancer pain patients (n = 434) receiving slow release oral morphine, excluding patients receiving opioids for only one day.

**Regressor**	**Adjusted Odds Ratio**[a] (95% CI)		**Nested model P-value**[b]
**Iceland treatment centre**[c]	0.38	(0.22 to 0.66)	0.0009
**Denmark treatment centre**[d]	0.13	(0.04 to 0.45)	0.002
TLR2 **rs3804100 carrier**[e,f]	0.42	(0.22 to 0.81)	0.01

[a]Odds Ratio controlling for all other regressors. Odds ratio less than 1 indicates an association with decreased likelihood of sickness response. [b]Likelihood ratio chi-square test P-value testing each term after all others (i.e. nested model comparisons) according to the marginality principle [Fox, J, Weisberg, S. An R Companion to Applied Regression. Third ed. Thousand Oaks, CA: Sage; 2019]. Reference groups are [c]non-Iceland treatment centre, [d]non-Denmark treatment centre and [e]homozygous wildtype genotype. [f]Carrier: heterozygous or homozygous variant.

Supplementary Table S10. Variables associated with opioid adverse event complaint in cancer pain patients (n = 434) receiving slow-release oral morphine (alternative model), excluding patients receiving opioids for only one day.

**Regressor**	**Adjusted Odds Ratio[a]** (95% CI)		**Nested model P-value[b]**
**Iceland treatment centre[c]**	0.37	(0.21 to 0.66)	0.0008
**Denmark treatment centre[d]**	0.14	(0.04 to 0.47)	0.002
***TLR2* rs3804100 variant carrier[e,g]**	0.43	(0.22 to 0.83)	0.01
***CASP1* rs554344 C/C[f]**	4.88	(0.98 to 88.5)	0.05

[a]Odds Ratio controlling for all other regressors. Odds ratio less than 1 indicates an association with decreased likelihood of sickness response. [b]Likelihood ratio chi-square test P-value testing each term after all others (i.e. nested model comparisons) according to the marginality principle [Fox, J, Weisberg, S. An R Companion to Applied Regression. Third ed. Thousand Oaks, CA: Sage; 2019]. Reference groups are [c]non-Iceland treatment centre, [d]non-Denmark treatment centre, [e]homozygous wildtype genotype and [f]homozygous wildtype or heterozygous genotype. [g]Carrier: heterozygous or homozygous variant.

Supplementary Table S11. SNP allele frequencies within each treatment centre country in EPOS cancer pain patients receiving slow-release oral morphine.

Gene	SNP	Nucleotide change	Variant allele frequency							
			**Swi** (n=44-47)	**Ger** (n=61-65)	**Den** (n=11-12)	**UK** (n=52-59)	**Ice** (n=89-91)	**Ita** (n=28-29)	**Nor** (n=146-156)	**Swe** (n=37-39)
IL6	rs10499563	T>C	0.17	0.21	0.33	0.18	0.18	0.31	0.17	0.18
IL1B	rs1143627	T>C	0.38	0.26	0.46	0.35	0.3	0.38	0.35	0.31
IL1B	rs16944	C>T	0.38	0.26	0.46	0.35	0.3	0.38	0.35	0.31
IL1B	rs1143634	C>T	0.16	0.27	0.17	0.17	0.29	0.18	0.24	0.22
LY96	rs11466004	C>T	0.02	0.02	0	0.02	0.01	0.07	0.02	0.01
OPRM1	rs1799971	A>G	0.13	0.12	0.17	0.16	0.12	0.26	0.13	0.08
TGFB1	rs11466314	G>A	0	0	0	0	0	0	0	0
TGFB1	rs1800469	C>T	0.26	0.32	0.29	0.24	0.26	0.52	0.29	0.38
TNFa	rs1800629	G>A	0.17	0.16	0.04	0.25	0.14	0.12	0.2	0.15
IL10	rs1800871	C>T	0.23	0.23	0.21	0.25	0.19	0.21	0.29	0.22
IL10	rs1800896	G>A	0.46	0.56	0.58	0.52	0.43	0.53	0.58	0.49
IL2	rs2069762	T>G	0.27	0.32	0.42	0.33	0.3	0.43	0.26	0.21
CRP	rs2794521	T>C	0.36	0.25	0.21	0.26	0.3	0.29	0.26	0.25
TLR2	rs3804100	T>C	0.07	0.1	0.04	0.06	0.05	0.05	0.07	0.06
TLR4	rs4986790	A>G	0.02	0.05	0.08	0.04	0.07	0.02	0.03	0.08
TLR4	rs4986791	C>T	0.02	0.05	0.08	0.04	0.07	0.02	0.03	0.06
CASP1	rs554344	G>C	0.16	0.27	0.04	0.21	0.23	0.12	0.17	0.18
CASP1	rs580253	G>A	0.16	0.27	0.04	0.21	0.22	0.12	0.17	0.17
BDNF	rs6265	G>A	0.21	0.22	0.25	0.19	0.14	0.29	0.16	0.27
MYD88	rs6853	A>G	0.14	0.14	0.17	0.14	0.12	0.12	0.1	0.13
IL6R	rs8192284	A>C	0.49	0.42	0.38	0.52	0.43	0.33	0.36	0.38
COMT	rs4680	G>A	0.5	0.5	0.62	0.53	0.53	0.43	0.56	0.54
ARRB2	rs3786047	A>G	0.7	0.66	0.58	0.68	0.68	0.78	0.65	0.71
ARRB2	rs1045280	C>T	0.68	0.65	0.59	0.67	0.7	0.78	0.64	0.71
ARRB2	rs2271167	A>G	0.68	0.66	0.58	0.66	0.7	0.78	0.64	0.69
ARRB2	rs2036657	G>A	0.69	0.65	0.58	0.67	0.7	0.78	0.64	0.71
ABCB1	rs1045642	G>A	0.59	0.49	0.54	0.49	0.62	0.53	0.54	0.62
ABCB1	rs2235013		0.49	0.53	0.46	0.55	0.38	0.53	0.51	0.4
ABCB1	rs1128503	G>A	0.49	0.39	0.5	0.4	0.47	0.45	0.41	0.55

Swi: Switzerland. Ger: Germany. Den: Denmark. UK: United Kingdom. Ice: Iceland. Ita: Italy. Nor: Norway. Swe: Sweden.
