# Supplementary material for: Pharmacokinetic and neuroimmune pharmacogenetic impacts on slow-release morphine cancer pain control and adverse effects
Source: Pharmacogenomics J. 2024 Jun 1;24(3):18. doi: 10.1038/s41397-024-00339-w (PMC11144121; doi:10.1038/s41397-024-00339-w)
Supplement: Supplementary file 4 — Supplementary Figures [file 41397_2024_339_MOESM4_ESM.pdf]

Supplementary Figures

This file contains Supplementary Figures for the manuscript entitled "Pharmacokinetic and neuroimmune pharmacogenetic impacts on slow-release morphine cancer pain control and adverse effects".

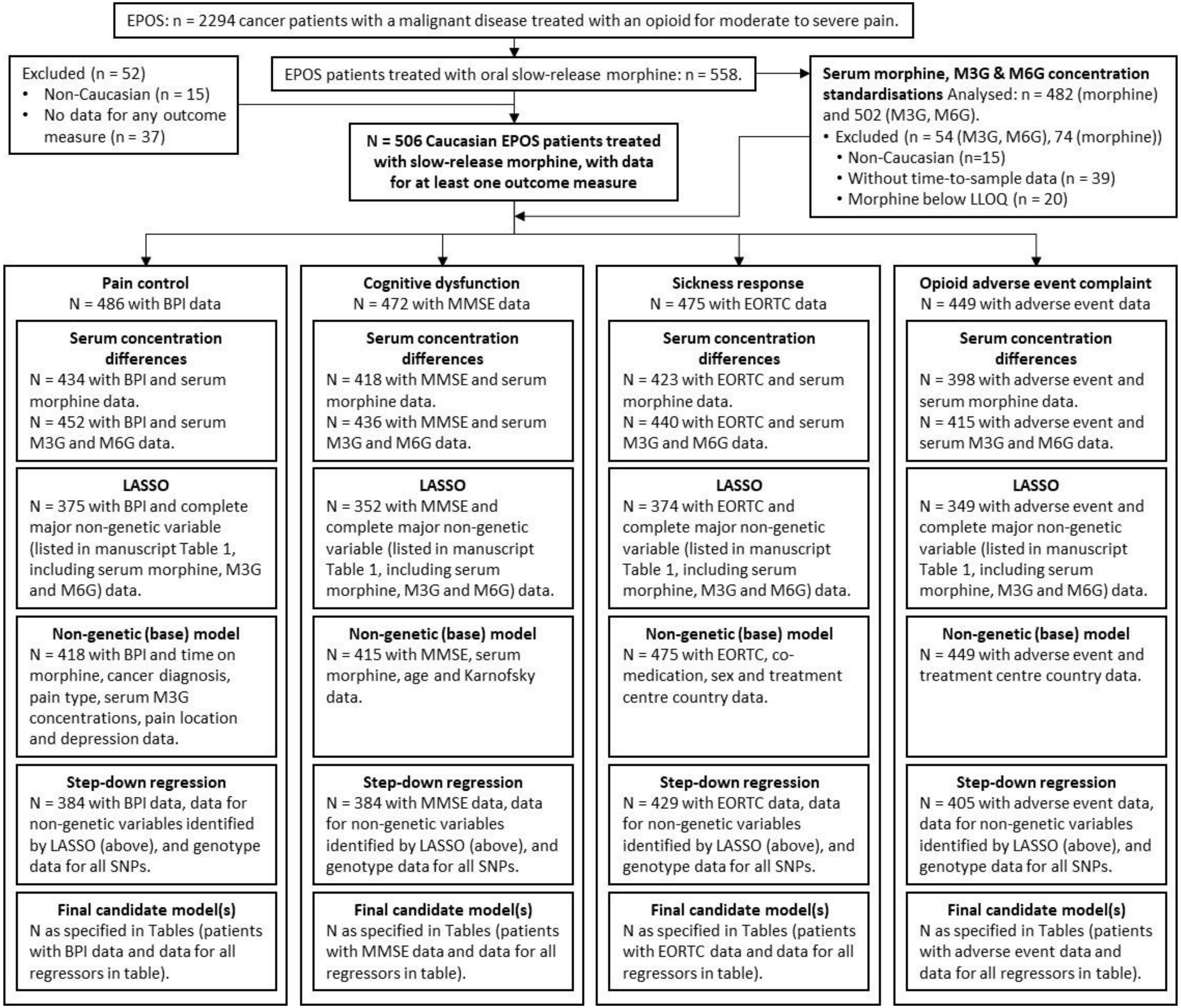

Supplementary Figure S1. Flowchart of the selection and exclusion criteria and resulting sample sizes for each analysis.

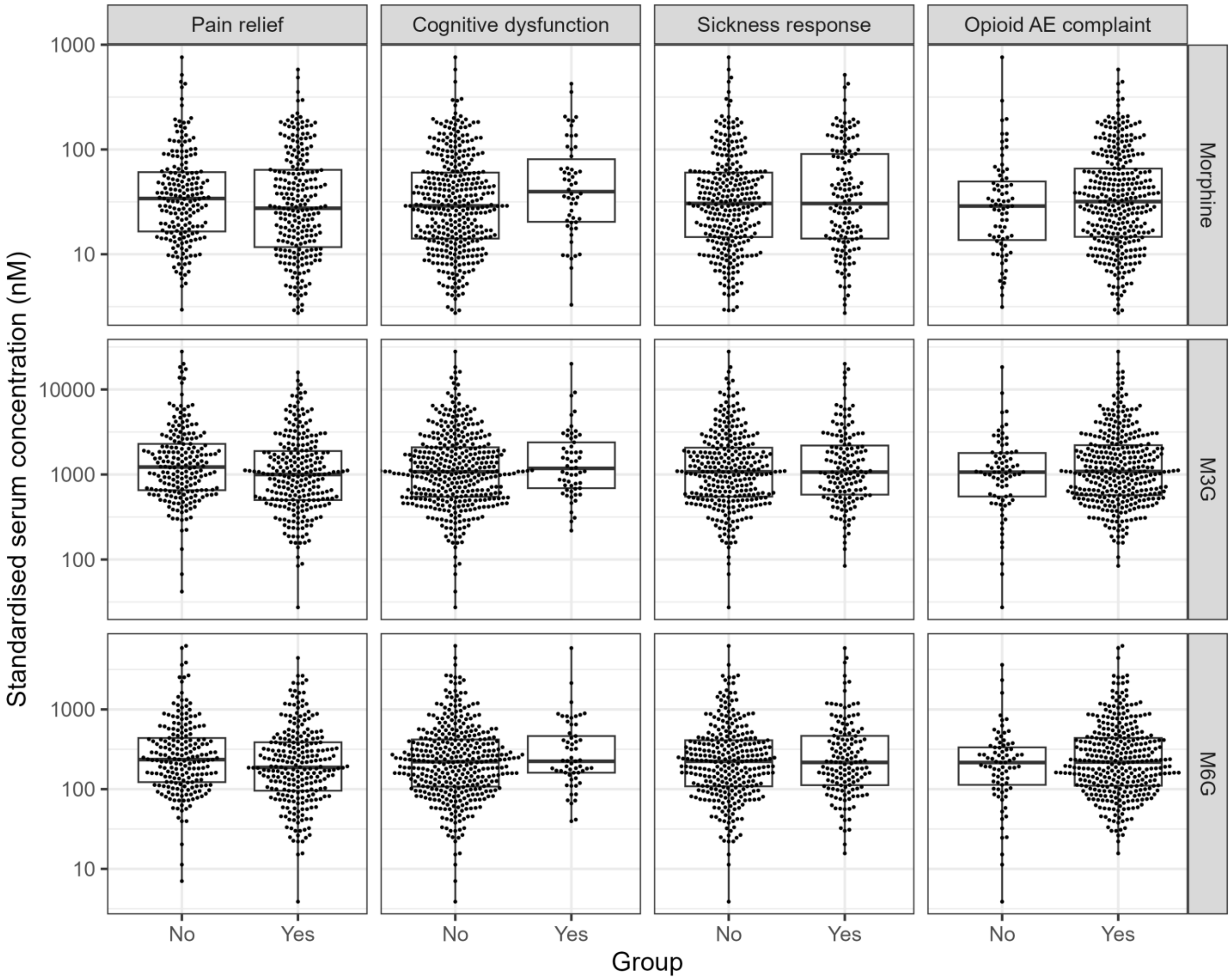

Supplementary Figure S2. Standardised serum morphine, M3G and M6G concentrations of cancer pain patients receiving slow-release oral morphine with or without pain relief, cognitive dysfunction, sickness response and opioid adverse event (AE) complaint.
